# Automatic detection and classification of manufacturing defects in metal boxes using deep neural networks

**DOI:** 10.1371/journal.pone.0203192

**Published:** 2018-11-09

**Authors:** Oumayma Essid, Hamid Laga, Chafik Samir

**Affiliations:** 1 CNRS LIMOS UMR 6158, University of Clermont Auvergne, France; 2 School of Engineering and Information Technology, Murdoch University, Perth, Australia; 3 Phenomics and Bioinformatics Research Centre, University of South Australia, Australia; Nanjing University of Information Science and Technology, CHINA

## Abstract

This paper develops a new machine vision framework for efficient detection and classification of manufacturing defects in metal boxes. Previous techniques, which are based on either visual inspection or on hand-crafted features, are both inaccurate and time consuming. In this paper, we show that by using autoencoder deep neural network (DNN) architecture, we are able to not only classify manufacturing defects, but also localize them with high accuracy. Compared to traditional techniques, DNNs are able to learn, in a supervised manner, the visual features that achieve the best performance. Our experiments on a database of real images demonstrate that our approach overcomes the state-of-the-art while remaining computationally competitive.

## 1 Introduction

Automatic inspection and defect detection using image processing is an area of machine vision that is being widely adopted in many industries. It is used for high throughput quality control in production systems such as the detection of flaws on manufactured surfaces, e.g. metallic rails [1] or steel surfaces [2]. The idea is to design autonomous devices that automatically detect and examine specific visual patterns from images and videos in order to overcome the limitations and improve the performance of the traditional inspection systems that depend heavily on human inspectors.

Various systems have been previously developed for the automatic inspection of surfaces of different products. They are generally composed of a pipeline of several steps, each one introduces a set of challenges. First, the acquisition step requires efficient calibration of various types of sensors such as cameras and lighting systems [3, 4]. The quality of the images produced by the acquisition systems impacts directly the performance of the subsequent analysis steps.

Once images are acquired, the second step is the extraction of visual features, which are then used as input to classifiers, which return the likelihood of the presence of a defect at each pixel of the image. These classifiers can be supervised or non-supervised. The main challenge here is two-fold; first one has to design features that are suitable for the problem at hand. The second one is the design of classifiers that are able to learn the boundaries in the feature space that separate defects from non-defects. While both problems have been extensively investigated [5, 6], existing techniques still fail to achieve high performance especially under challenging conditions such as those present in industrial environments.

This paper is concerned with the design of deep learning techniques that are able to automatically detect and classify manufacturing defects in the surface of metal boxes. The advantage of using deep learning techniques is their ability to learn, in a supervised manner, the visual features that achieve the best detection and classification performance. We show in the experimental part that the proposed framework outperforms traditional techniques that are based on hand-crafted features.

Real time defect detection, using image processing, has been extensively studied in the literature [7, 8]. Previous techniques can be divided into three general categories based on (1) their input (single vs. multiple images, RGB vs. depth images), (2) the type of features they extract from the input images, and (3) the type of classifiers they use for detecting defect regions and for classifying various types of defects. Ideally, one would like to detect and classify defects only from one single RGB image. However, this is not often an easy task especially when dealing with complex defects [9].

Once images are acquired, the next step is to extract visual features from them which will serve as input to some classifiers. Shen et al. [10], for example, segment images into regions, by grouping pixels of same intensity, and use some thresholding techniques to extract visual features. Shanmugamani et al. [9] used multiple textural features based on histogram and gray level co-occurrence combined with several classifiers based on Bayes, K-Nearest Neighbors (KNN), Artificial Neural Network (ANN), and Support Vector Machines (SVM) for detecting and classifying defects.

Once the features have been extracted, the last step is to learn a decision function, which returns the likelihood that a given feature corresponds to a defect region of a certain type. Such decision function is usually the output of a supervised or unsupervised classifier. Although several classifiers have been used, many previous works suggest that Support Vector Machines (SVM) [9] achieve better performance compared to Bayesian classifiers or Artificial Neural Networks (ANN) [5], especially when the classes that represent the various defects are separable.

While this widely used pipeline achieves satisfactory results, the main challenge in manufacturing systems is to achieve higher accuracies since improving the classification accuracy even with small percentages, can result in substantial economic gains. In fact, major advances can be achieved by letting the algorithms learn the discriminative features that optimally represent the data. This concept lies at the basis of many deep learning algorithms, which are models (networks) composed of many layers that transform input data (e.g. images) to outputs (e.g. binary decision true/false) while learning increasingly higher level features.

Convolutional Neural Networks (CNN) are one example of successful types of deep learning models. CNNs are composed of many layers. Each layer transforms its input by applying small convolution filters whose weights are learned in a supervised manner using large training samples. Despite their reliance on large training sets, deep learning has recently started delivering interesting results and showing impact in many applications [11, 12]. This is mainly due to the availability of large training datasets and powerful hardware that can accelerate the training process. The choice of a neural architecture depends on the application at hand. The most popular algorithms of deep learning are autoencoders [5, 11], deep belief networks, and convolutional neural networks [5, 13].

Since the first use of classical CNNs in inspection task [8, 14], they have shown a good classification accuracy for many applications such as the detection of defects on photometric stereo images of rail surfaces [1]. Another application of CNNs is the classification of steel images and used Pyramid of Histograms of Orientation Gradients (HOG) as feature extractor and Max-Pooling Convolutional Neural Networks. The approach achieves a high accuracy with 7% error rate, which is significantly better than other classifier such SVM [2].

Pretrained deep Convolutional Neural Networks have also achieved an excellent performance in image classification and recognition tasks. For instance, Kasthurirangan et al. [15] used pretrained CNN architectures to classify defects in crack pavement. First, some preprocessing methods were applied on the image dataset to enhance the information in the image. The pretrained VGG16 was used to extract, from images, features that can distinguish one image class from another. Different classifiers such as single-layer Neural Network (NN), SVM and Random Forest (RF) were tested proving the performance of (CNN) in this inspection task.

Autoencoder architectures have proved their efficiency with multi-modal data in many classification or retrieval tasks [11]. Their successors, stacked autoencoders [16], where each autoencoder is trained with the output of the hidden layer of the previous autoencoder in the stack. With this architecture, the level of complexity and abstraction of learned representation increase along the stack of autoencoders. Autoencoder trees, in analogy to decision trees, are a new form of neural networks that learn hierarchical representations at different resolutions where layers are replaced by tree’s nodes [17, 18].

In this paper, we propose a framework for detecting defects on metal boxes and for classifying images into defective or non-defective. The proposed framework uses Deep Neural Networks, which can learn, in a supervised manner, the appropriate features (and thus image representation) as well as the classification function. First, we apply some preprocessing techniques on images of a metallic box to improve their quality. Then, we use an adapted method based on a multi-layer network for learning. Recall that using an autoencoder leads to dimensionality reduction since the number of neurons in hidden layers is smaller than that of input layer. The classification decision is made using a probabilistic model that takes the output of the last layer and returns the likelihood of the images being of a defected metallic box. Finally, we also propose an algorithm that detects the defect if present in the image.

## 2 Material and methods

### 1 Data acquisition

**Fig 1:**
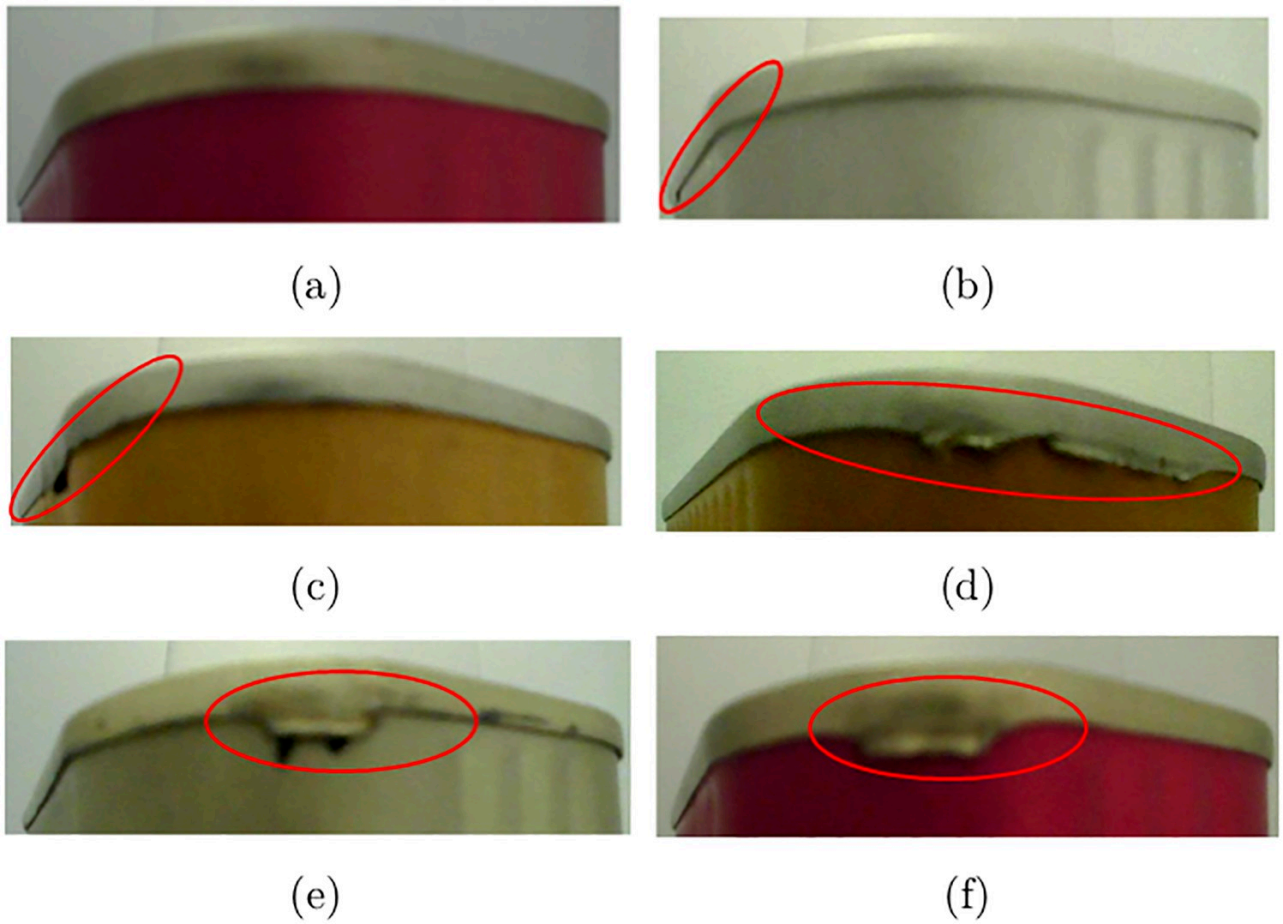
Examples of images: (a) without defect, (b)& (c) with small defects, and (d:f) with big defects.

Metallic boxes or steel can are extensively exploited as containers for the distribution or storage of food. The metallic boxes used in this study were collected by a company who produces steel cans with or without colored printing. Collected metallic boxes are suitable for packing tuna, salmon, fish, shrimp, mushroom, and other foodstuffs. All images were acquired from an installed and configured image acquisition device using Raspberry Pi camera module and processed by OpenCV in unconstrained environment on the company’s premises. For each metallic box, the device captures and stores two images that cover the entire box, see Fig 1. Such images can correspond to either non-defective (Fig 1(a)) or defective boxes. The defects can be small (Fig 1(b) and 1(c)) or big (Fig 1(d), 1(e) and 1(f)). In this paper, we consider that we have two different classes of images: defective and non-defective images.

### 2 Data description and representation

Due to non-controlled external factors, each step in the acquisition process may introduce noise (random changes) to the raw data (e.g. pixel values). To reduce the effects of such noise on the quality of the inspection process, we first pre-process the images by applying a set of denoising filters and reduce the spread by treating the database as a normal distribution of intensities [19, 20]. We refer to this step as *the image normalization process*. Another motivation for image normalization is to standardize the input of the autoencoder, used in the learning process, in order to reduce variability between intensity distributions [21].

**Fig 2:**
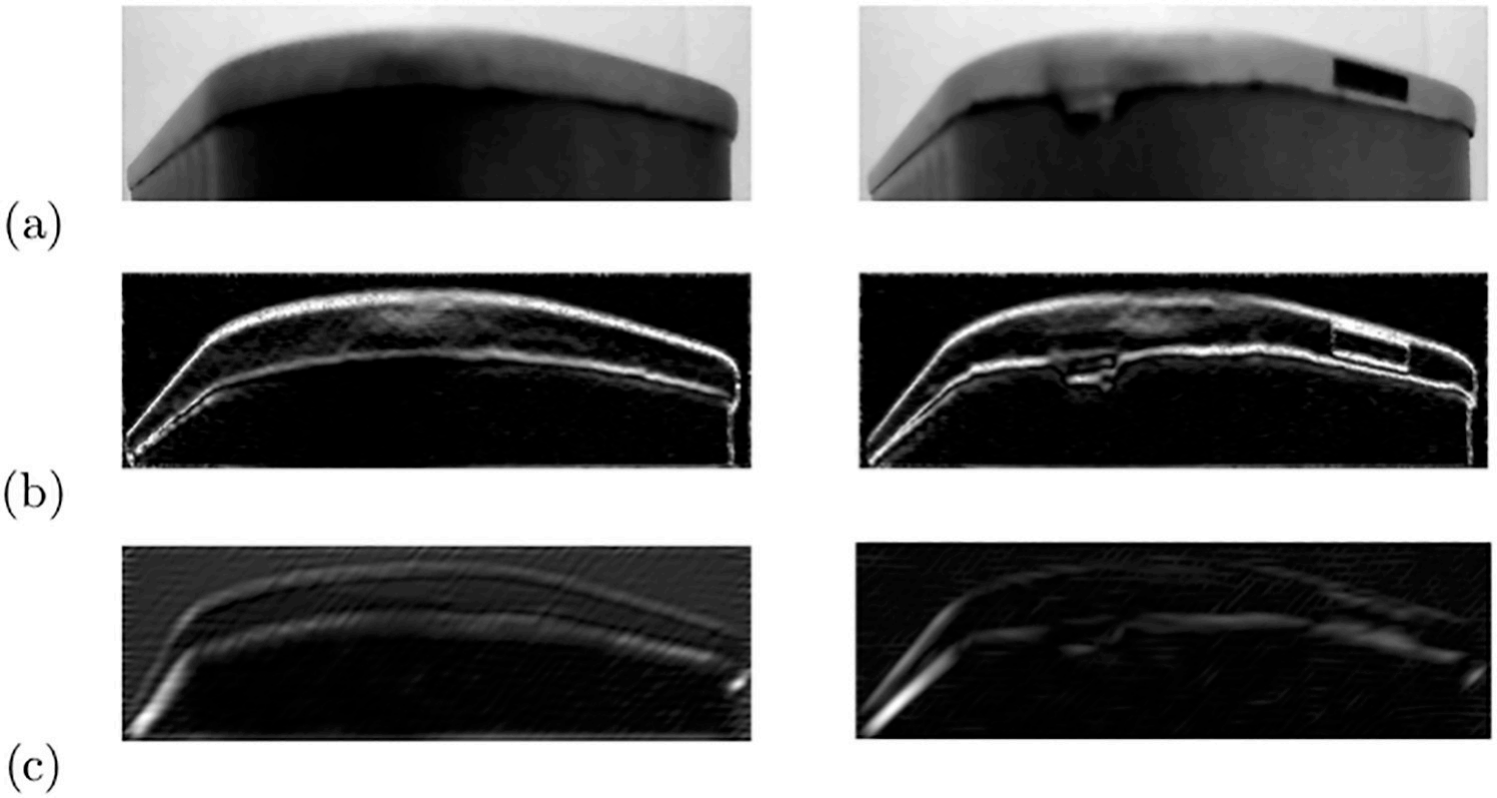
Examples of features extracted from two different images: (a) original images, (b) gradient, and (c) Gabor features.

Next, we observe that most of the defects are localized in areas that are dominated by horizontal edges, see Fig 1 for some examples. To highlight these regions, we use the gradient of the images instead of the images themselves [22]. Other representations and features have been proposed in the literature and we will use some of them for evaluation in our experiments. Since we are interested in horizontal contours where most of the defects are localized, we extract features by applying a Gabor filter with a $\frac{\pi }{2}$ orientation. Fig 2 shows two examples of extracted features where the first row shows the input images, the second and the third rows show the gradient and the Gabor features, respectively. Another way to capture relevant features is to decompose the image using adaptive basis such as Fourier, wavelets, or polynomials. Wavelet decompositions have been successfully used for many tasks such as dimensionality reduction, image/video classification, and many other areas of image analysis [23]. This motivated their use for evaluation in our experiments which has shown to be successful.

### 3 Features learning using an autoencoder

An autoencoder neural network architecture is a feedforward network composed of one or multiple connected hidden layers. It uses a non linear mapping function between the original data as input and outputs specific learned features [24, 25]. Several previous works have used autoencoders for feature learning or for dimensionality reduction since the number of neurons in the hidden layer is smaller than the dimension of the input. In fact, many studies [26, 27] showed that an autoencoder with nonlinear characteristic is more efficient than linear dimensionality reduction techniques such as Principal Component Analysis (PCA) when the input data have a nonlinear or a sparse structure. Overall, this provides significant benefits for the inspection task: the computational time and the required memory for storage. And even better, removing the redundant information by maximizing the covariance.

Stacked autoencoders [16, 28] are deep neural architectures composed of a succession of autoencoders. Each autoencoder is trained with the output of the hidden layer of the previous autoencoder in the stack. This architecture, which can be seen as a convolution, allows learning complex concepts in an progressive manner. Consequently, the output representations are more relevant when one autoencoder is not sufficient to capture interesting structures that maximize the covariance between the components.

In this work, we will follow the same strategy; We train an autoencoder with different layers to reduce dimensionality as well as extracting relevant features under constraints formulated by a cost function. In the training phase, we used a sparse autoencoder whose training criterion involves a sparsity penalty. Given a finite set of *N* images, represented by vectors *x*_*i*_ with *i* = 1…*N*, and their corresponding labels *y*_*i*_ ∈ {0, 1}, the learning is done by stacking multiple layers and the cost function *E* is defined by:

$$\begin{array}{c} E\left(X,Y,W,b\right)=\frac{1-\lambda }{2}∥Y-r\left(X,W,b\right){∥}_{2}^{2}+\frac{\lambda }{2}∥W{∥}_{2}^{2}, \end{array}$$ (1)

where *r* is the activation function, *X* and *Y* represent the observations, *W* and *b* are the network parameters, and ${∥W∥}_{2}^{2}$ is a regularization term. The Lagrangian parameter λ weights between bounds of *W* and proximity to the input.

This cost function is a deterministic functional on parametric functions of the form *r*(*X*) = *W*^*T*^ *X* + *b* that can be minimized using numerical methods to search for optimal parameters $\hat{W}$ and $\hat{b}$. Focusing on neural networks, a wide variety of models can be learned by varying different factors such as the activation function, the number of hidden units, and the choice of the regularization term. The details of the parameters initialization and the optimization process have been discussed and studied in the literature without determining the best choices. We consider that the answer is out of the scope of this paper and we give details of our model in Section 3.

One important aspect about autoencoders is dimensionality reduction and the features computed at the hidden layers, which are useful for inspection tasks. If the output of the autoencoder is considered as a deterministic function, one can plug it into a probabilistic model and perform learning and regression within the same framework. This idea is the main motivation of the proposed method and we will show that it helps improving the performance of the classification.

**Fig 3:**
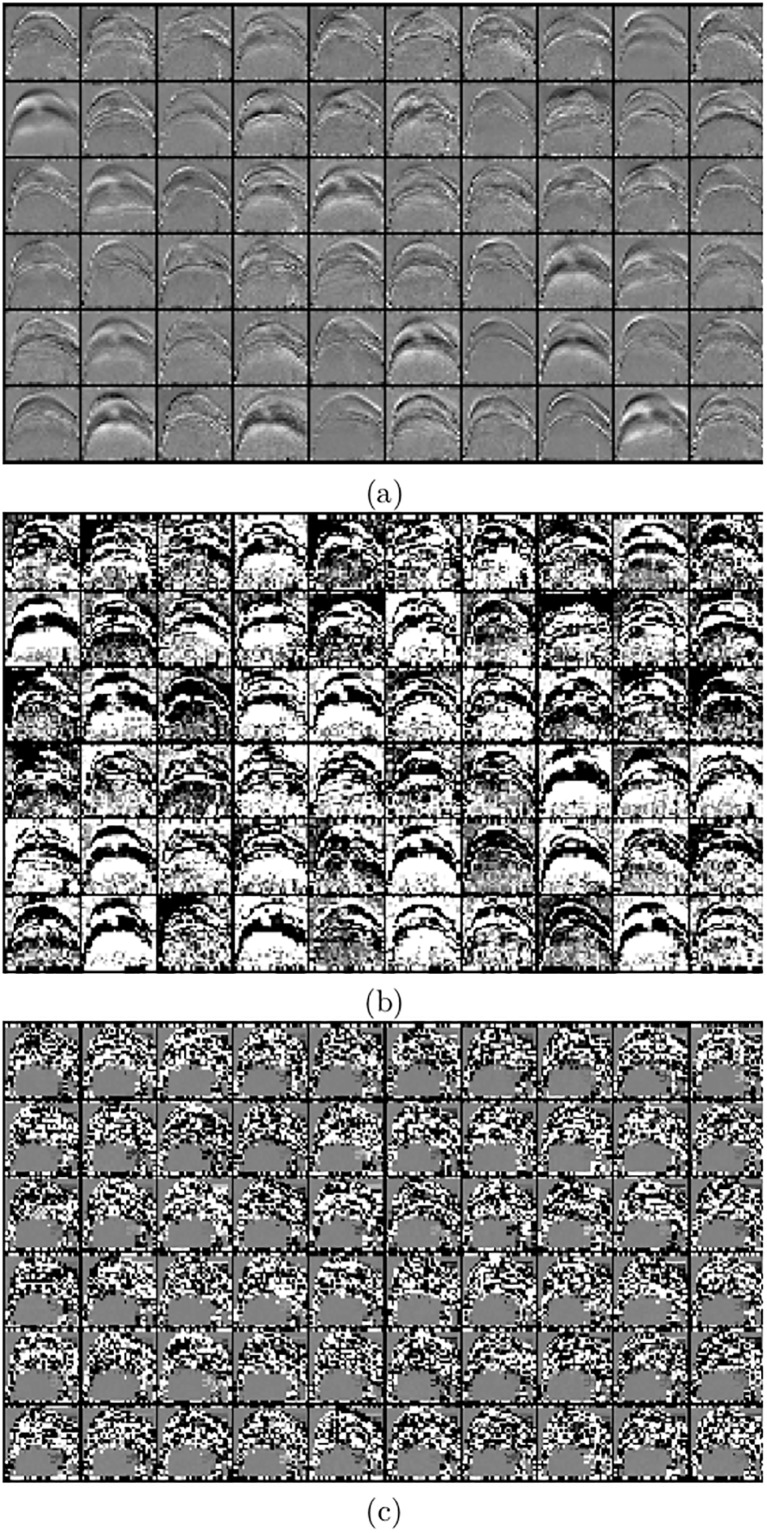
Visualization of the output features from the second hidden layer when applying an autoencoder on: (a) grayscale images, (b) gradient images, and (c) the binary images.

The idea of learning relevant features from data automatically as an output of an autoencoder can be illustrated by visualizing the hidden layers. For an autoencoder with several layers, every layer encodes different features. For example, Fig 3 shows (a) the output of the second hidden layer of the autoencoder trained on a set of grayscale images, (b) their corresponding gradients, and (c) gradient after binarization. Note that at this stage, the classifier is not yet defined but we expect the output to highlight relevant features. Even if it is hard to judge which one is better, we can say that the area around contours is further enhanced for different inputs.

### 4 Regression using Gaussian processes

We assume that we have *N* observations (*X*_1_, *Y*_1_),‥,(*X*_*N*_, *Y*_*N*_) of the same law of variables (*X*, *Y*). Here *X* is the observation (e.g. input image) and *Y* is its label. To be more specific, we restrict our analysis to the cases where *X* has a compact support in a finite-dimensional Euclidean space and *Y* is the class label with *y* = 0 for non-defective boxes and *y* = 1 for defective boxes.

Any regression model is based on building a predictive model: learn a probabilistic model from observed *Y* at different locations of *X* that will be able to predict y*, the class label, of a new vector x*. Before giving details of the proposed model, we first review classical regression methods, which consider *X* as elements on a high dimensional space. Then we provide a brief introduction to dimensionality reduction techniques. Next, we introduce the automatic learned features from an autoencoder as a parametric transformation of the input. Finally, we give details of Manifold Regression, the proposed method for jointly learning a regression model and a suitable feature representation *ϕ* of the data. Though mildly technical, it is useful as we focus on a particular subset of the relevant background in forming our model developments. We begin by reviewing a logistic regression model that has been initially introduced in [29]. The goal is to find the best fitting model representing the relationship between the output variable *Y* and a set of input variable *X* = (*X*_1_,‥, *X*_*p*_) where *Y* ∈ {0, 1}.

#### 4.1 MLE-based regression

We suppose that we have *N* mutually independent samples (*X*_1_, *Y*_1_),‥,(*X*_*N*_, *Y*_*N*_) of the same law as (*X*, *Y*). We consider the problem of fitting a logistic model *y* = *σ*(*x*^*T*^ *β*) where *σ* is the sigmoid function which is equivalent to estimating *β* from observed samples. Let (*x*_*i*_, *y*_*i*_) be the observed value of the explanatory variables (*X*_*i*_, *Y*_*i*_) for each observation *i* ∈ {1,‥, *N*}. We denote by *π*_*β*_(*x*_*i*_) the probability of *Y*_*i*_ = 1 for a given *X* = *x*_*i*_:

$$\begin{array}{c} P\left(Y_{i}=1|X=x_{i}\right)=\pi_{\beta }\left(x_{i}\right)=\frac{\exp({x_{i}}^{T}\beta )}{1+\exp({x_{i}}^{T}\beta )} \end{array}$$ (2)

and by modeling *Y*_*i*_|*X* = *x*_*i*_ as a Bernoulli distribution *π*_*β*_(*x*_*i*_), $Y_{i}|X=x_{i}∼B\left(\pi_{\beta }\left(x_{i}\right)\right)$ we write the negative log-likelihood as:

$$\begin{array}{ccc} l(\beta ) & = & \sum_{i=1}^{N}y_{i}\log\left(\pi_{\beta }\left(x_{i}\right)\right)+\left(1-y_{i}\right)\log\left(1-\pi_{\beta }\left(x_{i}\right)\right) \end{array}$$ (3)

$$\begin{array}{ccc} & = & \sum_{i=1}^{N}y_{i}{x_{i}}^{T}\beta -\log\left(1+\exp\left({x_{i}}^{T}\beta \right)\right) \end{array}$$ (4)

Then the gradient of *l* at *β* is given by:

$$\begin{array}{ccc} \nabla l(\beta ) & = & \sum_{i=1}^{N}x_{i}\left(y_{i}-\pi_{\beta }\left(x_{i}\right)\right) \end{array}$$ (5)

$$\begin{array}{ccc} & = & X^{T}\left(Y-\pi_{\beta }\right) \end{array}$$ (6)

where *π*_*β*_ = (*π*_*β*_(*x*_1_),‥, *π*_*β*_(*x*_*N*_))^*T*^, *X* = (*x*_1_,‥, *x*_*N*_)^*T*^ and *Y* = (*y*_1_,‥, *y*_*N*_)^*T*^. Typically, to find the MLE we have to search for the critical point $\hat{\beta }$ of the gradient: $\nabla l(\hat{\beta })=0$. Then the maximization of *l*(*β*) yields to the ordinary MLE β*. If *X* is of maximum rank, we have *β* ↦ *l*(*β*) is strictly concave, i.e β* exists and is unique [29]. We note that finding β* explicitly is not straightforward, and consequently is very common to use iterative algorithms based on Newton and gradient descent methods.

#### 4.2 Penalized MLE-based regression

Basically inspired from the Ridge logistic [29], the weighted logistic method is more suitable when the components of *X* are highly correlated or when we have a large number of explanatory variables. Remind that in both cases *X* is not of maximum rank which means that there is no guarantee for β* to exist nor to be unique. In an effort to address this issue, the idea of ridge logistic consists in adding a regularization term. The regularization will have the effect of controlling the model and improving the performance in the presence of an over-fitting. Consequently, we consider a modified version of Eq 5.

$$\begin{array}{c} l^{\lambda }\left(\beta \right)=\frac{\left(1-\lambda \right)}{2}l\left(\beta \right)-\frac{\lambda }{2}{\left||\beta |\right|}_{2}^{2} \end{array}$$ (7)

where the regularization parameter satisfies: 0 < λ < 1. We denote by *β*^λ,*^ the optimal solution. Therefore for a better choice of λ, the estimator *β*^λ,*^ should maximize the log-likelihood compared to the unstructured MLE, ie. MSE(*β*^λ,*^) < MSE(β*). Following the same steps for the new modified cost, the gradient of *l*^λ^(*β*) is:

$$\begin{array}{c} \nabla l^{\lambda }\left(\beta \right)=\frac{\left(1-\lambda \right)}{2}\nabla l\left(\beta \right)-\lambda \beta \end{array}$$ (8)

with ∇*l*(*β*) is the gradient of *l*(*β*) as detailed in Eq 5. Then the estimator is a solution of ∇*l*^λ^(*β*) = 0 and the negative Hessian of *l*^λ^(*β*) is

$$\begin{array}{c} H^{\lambda }\left(\beta \right)=\frac{\left(1-\lambda \right)}{2}H\left(\beta \right)+\lambda I \end{array}$$ (9)

with

$$\begin{array}{c} H\left(\beta \right)=-\sum_{i=1}^{N}x_{i}x_{i}^{T}\pi_{\beta }\left(x_{i}\right)\left(1-\pi_{\beta }\left(x_{i}\right)\right) \end{array}$$ (10)

As for the previous optimization formulation we use an iterative approach based on Newton method. For numerical efficiency, we present an approximation of the Newton-MLE based on Taylor expansion of ∇*l*^λ^(*β*^1^) at *β*^0^:

$$\begin{array}{c} \nabla l^{\lambda }\left(\beta^{1}\right)\approx \nabla l^{\lambda }\left(\beta^{0}\right)-{\left(\beta^{1}-\beta^{0}\right)}^{T}H^{\lambda }\left(\beta^{0}\right) \end{array}$$ (11)

In particular, we have ∇*l*^λ^(*β*^1^) = 0 and a first-order approximation for *β*^1^ as:

$$\begin{array}{c} \beta^{1}\approx \beta^{0}+{\left(\frac{\left(1-\lambda \right)}{2}H^{\lambda }\left(\beta^{0}\right)+\lambda I\right)}^{-1}\left(\frac{\left(1-\lambda \right)}{2}\nabla l^{\lambda }\left(\beta^{0}\right)-\lambda \beta \right) \end{array}$$ (12)

This process is then updated iteratively until convergence.

$$\begin{array}{c} \beta^{k+1}\approx \frac{\left(1-\lambda \right)}{2}{\left(\frac{\left(1-\lambda \right)}{2}H\left(\beta^{k}\right)+\lambda I\right)}^{-1}\left(H\left(\beta^{k}\right)+\nabla l\left(\beta^{k}\right)\right) \end{array}$$ (13)

### 5 Gaussian processes classifier

In this section we make connection with binary classification, as described in the previous section, which forms the foundation of Gaussian processes (GP). Gaussian Processes are a state-of-the-art non-parametric probabilistic regression method. In order to capture the correlation between observed *X*s, build a probabilistic model and perform optimal predictions for non-observed data, we study a GP as a distribution over the transformed variables *ϕ* ∼ *GP*(*m*, *C*) and fully defined by a mean function *m* (in our case *m* = 0) and a covariance function *C*. The popularity of such processes stems primarily from two essential properties. First, a Gaussian process is completely determined by its mean and covariance functions. This property facilitates model fitting as only the first- and second-order moments of the process require specification. Second, solving the prediction problem is straightforward since the optimal predictor at an unobserved position is a linear function of the observed values.

In the simple case of a real random process, Y(x),x∈R is a Gaussian process if all the finite-dimensional distributions have a multivariate normal distribution. That is, for distinct observations *x*_1_, *x*_2_, …, *x*_*n*_, the random vector *Y*_1_, *Y*_2_, …, *Y*_*n*_ with *Y*_*i*_ = *Y*(*x*_*i*_) has a multivariate normal distribution with mean vector *m* = *E*[*Y*] and covariance matrix *C* with *C*_*i*,*j*_ = *C*(*Y*_*i*_, *Y*_*j*_). A Gaussian process is said to be stationary if *m* is independent of *x* and the covariance *C*(*Y*(*x*+*h*), *Y*(*x*)) = *C*(*h*) < ∞ is independent of *x* (i.e., the process *Y*(*x*) is translation invariant). This condition is usually called the strong form of stationarity (second-order or weak). Considering the new formulation, we introduce a new latent variable *ϕ* with:

$$\begin{array}{c} \pi =P(Y|X=x)=\sigma (\phi (x)) \end{array}$$ (14)

The idea behind GP prediction is based on placing a GP prior on *ϕ*, i.e: $\phi ∼GP(m,C_{\theta })$. Where *m* is the mean, equal to zero in our case, and *C*_*θ*_ is a family of covariance functions with parameter *θ*. There is a wide choice of covariance functions but, as in this study, the Matérn covariance functions is a preferred choice due its smoothness and asymptotic properties [30, 31]. If we consider prediction from the observed locations. Obviously, we are looking for a prediction method that works well on the average. One of the main difficulties is the choice of the covariance function. Despite the fact that a prediction model may yield to an unbiased predictor with a correct predictive variance, it is only true if the choice of a covariance function is optimal. To deal with such issue, a common choice consists in parametric covariance functions leading to an search for an optimal parameter $\hat{\theta }$. Many numerical methods have been used to search for $\hat{\theta }$ and the most studied and popular one is Maximum Likelihood Estimator (MLE).

We keep the same notations from the previous section and by abuse of notation, we note *ϕ* = (*ϕ*_1_,‥, *ϕ*_*N*_)^*T*^ = (*ϕ*(*x*_1_),‥, *ϕ*(*x*_*N*_))^*T*^. Therefore, the goal is to estimate the hyperparameter *θ* = (*α*, *τ*, *ν*) of the covariance function *C*_*θ*_ that minimizes the negative likelihood *L*_*θ*_:

$$\begin{array}{ccc} L_{N}\left(\theta |\phi \right) & = & \frac{N}{2\pi }\text{ln}\left(2\pi \right)+\frac{N}{2}\text{ln}\left(\tau \right)+\frac{1}{2\tau }\text{ln}\left(\text{det}\;C_{\theta }\right)+\frac{1}{2}\phi^{T}C_{\theta }^{-1}\phi \end{array}$$ (15)

where *C*_*θ*,*i*,*j*_ = *C*_*θ*_(*d*(*ϕ*_*i*_, *ϕ*_*j*_)) is the covariance matrix for *ϕ*_1_, *ϕ*_2_, …, *ϕ*_*n*_ and *θ* is the full parameter vector taking values in the parameter space Θ={τ>0,α>0,ν=1+k,k∈N}. Our goal is then to find the maximum likelihood estimator (MLE) $\hat{\theta }$ of *θ*. Once, there is no analytical solution for Eq 15, we use a Newton-based method to find the MLE. The optimization problem over *ν* is not straightforward in this case, and thus, we estimate this value using a *k*-fold cross-validation rather than the MLE.

To summarize, the GP predictive distribution at a new observation ϕ* = *ϕ*(X*) is given by

$$\hat{P}\left(\phi^{*}|X,Y,X^{*}\right)=N(\mu \left(X^{*}\right),\sigma^{2}\left(X^{*}\right))$$ (16)

$$\mu (X^{*})=c_{*}^{T}C^{-1}\hat{\phi }$$ (17)

$$\sigma^{2}\left(X^{*}\right)=c_{**}-c_{*}^{T}{\left(C+W^{-1}\right)}^{-1}c_{*}$$ (18)

where c_**_ = *C*(X*, X*) and c_*_ = *C*(*X*, X*) and *W* is a *N* × *N* diagonal matrix with $W_{ii}=\frac{\text{exp}(\phi_{i})}{1+\text{exp}(\phi_{i})}$. Given the mean and the variance, we make predictions by computing the conditional expectation:

$${\bar{\pi }}_{*}\approx E_{\hat{P}}\left(\pi_{*}|X,Y,X*\right)=\int \sigma \left(\phi *\right)\hat{P}\left(\phi *|X,Y,X*\right)d\phi *$$ (19)

## 3 Results

We evaluate the performance and efficiency of the proposed approach on a database of 2042 real images. The database contains 530 images of defective metallic boxes and 1512 images of non-defective ones. First, we will look at the ability of our approach to classify defective and non-defective images. Results demonstrate that the autoencoder can be successfully applied to learning relevant features from different inputs. Combined with the GP classifier, it reaches good performances. Second, we evaluate the proposed method for detecting and localizing defects in images. For both cases, we compare the performances of the autoencoder combined with the GP classifier using the Matérn covariance function [32], and the Newton method to search for the maximum likelihood estimator. We train the autoencoder with 75% of the images, i.e. 1531 images, 1148 with defects and 397 without defects. The rest of the images in the dataset is used for test. In order to remove the test bias, we use 100–fold cross validation, i.e. we run the method 100 times. At each run, we randomly select the training set (75% of the entire dataset) and the remaining 25% are used for test. We then average the performance over the 100 runs. To evaluate the classification quality of the different models, we consider the False Negative (**FN**) and False Positive (**FP**) rates where:

- FN rate corresponds to the number of images labeled as non-defective but classified as defective.
- FP rate corresponds to the number of images labeled as defective but classified as non-defective.

We compare the proposed method with other state-of-the-art methods using the same experimental protocol. We use different image representations combined with two classifiers: (1) the Matlab implementation of K-Nearest Neighbor classifier (KNN). We used the Euclidean distance for finding the nearest neighbors. (2) The Matlab implementation of the Support Vector Machine (SVM) classifier.

### Image classification

This section presents the performance of the proposed method when applied to classify images. In our implementation, we used a model which consists of two stacked autoencoders with 50 and 60 hidden layers, respectively. The sparsity penalty λ is set to 10^−4^ for all the experiments. The optimal parameters $\hat{W}$ and $\hat{b}$ were obtained by optimizing the cost using an iterative Newton-based method, initialized using a standard normal distribution.

There are three key steps that can affect the performance of any classification method: (i) The representation of the images and the definition of the feature space. (ii) The analysis of these observations in the feature spaces. (iii) The classifiers used to classify the observations. To illustrate their importance in the application context, we use two classifiers: the K-Nearest Neighbor (KNN) and the Support Vector Machines (SVM) classifiers, and six different features to represent each image:

1. Pre-processed image intensity.
2. Vertical gradient of (a).
3. Binarized version of (b).
4. Histogram of Oriented Gradients (HOG) computed from (a).
5. Coefficients from the decomposition of (a) into a linear combination of the Haar wavelet basis.
6. Gabor descriptor computed from (a) using two directions $\left\{\frac{\pi }{2},\frac{3\pi }{4}\right\}$

These features are then compared to the method proposed in this article, which can take any input as a feature vector. It then searches for the optimal parameters and features during the training stage, and predicts the correct label for test data. Experimental results show that the KNN classifier performs worse than SVM for all type of features. Compared to KNN, SVM shows better classification performances but remains below the proposed method. Table 1 summarizes the classification performance of these methods in terms of False Negative (FN) and False Positive (FP) rates.

**Table 1:** Classification performance with different features for representing images and different classifiers. The best performances are indicated in bold (the lower the better). These results are obtained using 100-fold cross validation.

Features	Rate	Classification Method		
		KNN	SVM	Autoencoder
**Grayscale**	FP	29.8%	29.4%	**25**%
	FN	28.5%	27%	**24.8**%
**Gradient**	FP	26.2%	24.5%	**23.4**%
	FN	29.8%	29.4%	**16.9**%
**Binary**	FP	25.9%	25%	**21.9**%
	FN	20.1%	15%	**13.2**%
**Gabor**	FP	38.2%	32%	**26.9**%
	FN	45%	38.3%	**34.8**%
**HOG**	FP	25%	19.2%	**19**%
	FN	18.9%	13.8%	**10**%
**Haar**	FP	24.2%	23%	**18.9**%
	FN	17%	14%	**7.9**%

According to Table 1, we note that the proposed method outperforms the other methods regardless of the features used to represent the images. In particular, we observe that the best accuracies are achieved when combining either Haar decomposition and / or HOG descriptors as input with SVM for classification or with our proposed autoencoder model.

**Fig 4:**
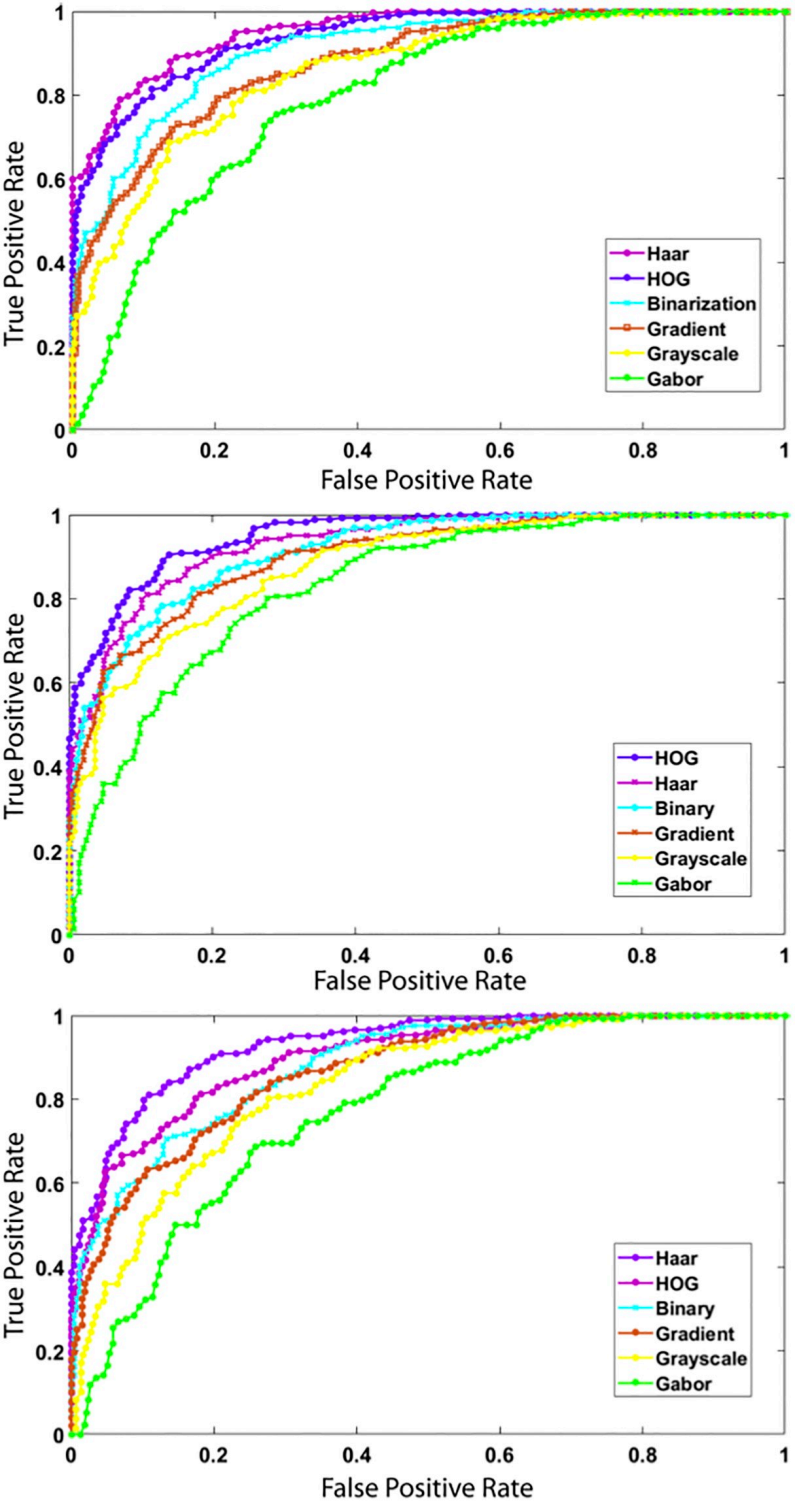
ROC curves of the methods with different images features and different classifiers: the proposed method (top), SVM (middle), and KNN (bottom).

The ROC curves of Fig 4 show that the proposed method has the most predictive power and generalization capability with a value of 0.86, followed by SVM 0.815 and then KNN 0.794. This indicates that the proposed method succeeds in learning relevant features, reducing dimensionally, and predicting more accurately.

### Detection and localization of defects

Finally, we extend the proposed approach to detecting and localizing defects in images. First, to build the training (and testing) data, we asked an expert to manually localize, in each input image, regions that contain defects. We then divided all the images into patches of size 32 × 32. Patches that contain defects are then treated as negative examples while the remaining are treated as positive examples. All the patches were subject to the same preprocessing step as the one used for the previous experiments. We selected HOG descriptors and Haar coefficients to represent an image since they achieved the best classification accuracy, see Table 1.

To show the effectiveness of our approach, we use the same database for classifying and detecting defective sub-regions. We also compare our method with HOG-SVM and Haar-SVM models, and a pre-trained CNNs such as VGG-16. VGG 16-layer is a deep convolutional neural network pretrained on ImageNet, a large image dataset composed of 1000 classes and 1.3M images [33]. We use the MatConvNet implementation [33] of VGG-16, with an additional fine-tuning step, combined with a softmax classifier.

**Fig 5:**
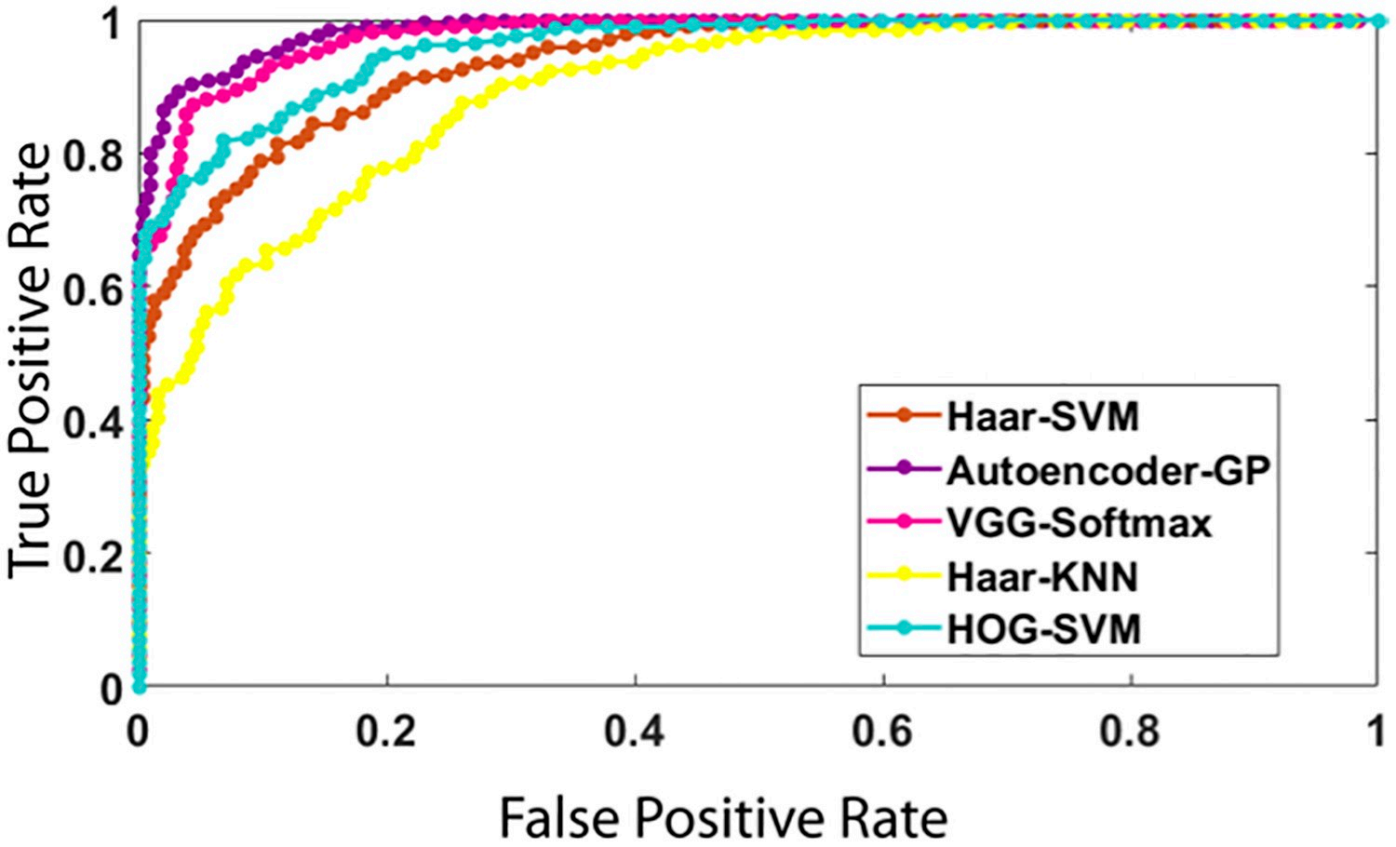
ROC curves of the methods with different features for representing images and different classifiers.

Table 2 summarizes the defects detection rates for all methods. These results are obtained using 100-fold cross validation using the same setup as the previous experiment. From Table 2, we observe that our approach outperforms the other methods. The ROC curves in Fig 5 confirm that both VGG-softmax and our model reach a good accuracy with a slight advantage for our method.

**Table 2:** Performance of the methods for detecting defective patches. Rates are obtained using 100-fold cross validation. Here, we provide the average performance and the standard deviation (Std) over the 100 runs.

Method	FP	Std	FN	Std
Haar-SVM	17%	0.98	12.2%	1.10
Haar-KNN	21.8%	1.5	16.5%	1
HOG-SVM	14.5%	1.1	10%	1.2
VGG-softmax	13.02%	1.95	8.6%	1.34
The proposed	**10.6**%	1.4	**5.41**%	1.39

**Fig 6:**
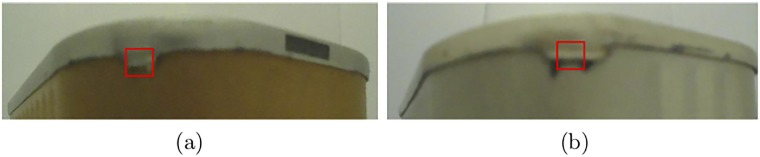
Examples of successful defects detections (a & b) using the proposed method.

Finally, Fig 6 shows examples of localized defects on the original test images.

## 4 Discussion

The new framework proposed in this article, which enables real inspection and classification of defects in metallic boxes, can be generalized for any application that deals with detecting non-standard subregions. The representation part, i.e. feature extraction, was used to illustrate the ability of our framework to capture discriminant information from the input. Other features from the literature could be also used or adapted for the application at hand. The investigation of the best representation is out of the scope of this work. Nevertheless, the features can be selected independently and then used following the same procedure described in this work. One can also use different features selection [34, 35] and similarity learning [36] methods to automatically select the features that achieve the best performance.

It addition to the accuracy of the defect detection results, the proposed framework has at least three advantages compared to the state-of-the-art:

- First, it provides more flexibility compared to pre-trained CNNs: our method can be adapted to the dimension of the input. In fact, it can accept input of any dimension and choose the output dimension by fixing the size of the different layers. This leads to a reduced dimension, which in turn improves the computational efficiency.
- The input can be a vector of any features or a mixture of them. This can lead to a different network architecture but the overall process remains the same.
- The classifier includes a mapping function which includes the non-linearity of data making this framework more general then linear classifiers.

It should be noted that there is still room for further improvements, especially when it comes to the prediction part where we used a Newton-bas method to search for optimal parameters of the Gaussian process. More sophisticated stochastic tools, such as MCMC, may improve the quality of the estimator.

**Fig 7:**
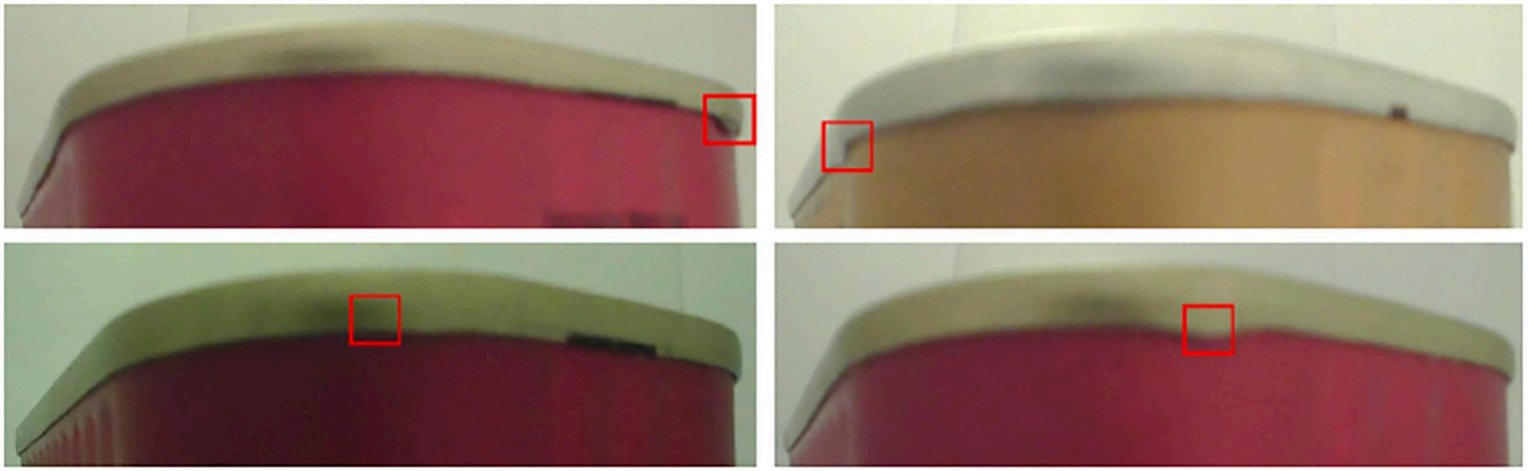
Examples of incorrect detections: False positive (top row) and false negative (bottom row).

Fig 7 highlights some failure cases (false positives and false negatives) of the proposed method. This figure shows that it is not always a straightforward decision whether a subregion contains a defect or not (see Fig 7 (top row)). In fact, some defects are very small and that images were manually classified by one expert. This means that it could be hard even for an expert to decide visually what is a defect when it is small or has a different shape (see Fig 7 (bottom row)). An extension to this work would be to ask different experts to label images independently and to learn uncertainty at the same time as labels. This will make the framework more complete but will need more complex methodologies to come even close to handling the hard challenges resulting from such formulations.

## 5 Conclusion

We proposed in this article a new machine learning method for detecting and localizing defects in images of metallic boxes. The proposed method is based on: (1) an autoencoder to automatically learn features from the input, and (2) a Gaussian process classifier. Different image representations were used as an input to the autoencoder and two of them were selected for detection: HOG descriptor and the decomposition in a wavelet basis. To show the effectiveness of our approach, we used the same database for classifying and detecting defective sub-regions. We have also compared our method with other state-of-the-art techniques, namely the HOG-SVM and the Haar-SVM models, and a pretrained CNNs such as VGG-16. The experimental results demonstrate that the proposed method achieves the best performance. It can be successfully applied to learning relevant features from different inputs and when combined with the GP classifier.

## Supporting information

S1 Dataset (ZIP)
